# Oxidative Stress and the Kidney in the Space Environment

**DOI:** 10.3390/ijms19103176

**Published:** 2018-10-15

**Authors:** Paraskevi Pavlakou, Evangelia Dounousi, Stefanos Roumeliotis, Theodoros Eleftheriadis, Vassilios Liakopoulos

**Affiliations:** 1 Department of Nephrology, Medical School, University of Ioannina, 45110 Ioannina, Greece; pavlakoup@gmail.com (P.P.); evangeldou@gmail.com (E.D.); 2 Division of Nephrology and Hypertension, 1st Department of Internal Medicine, AHEPA Hospital, School of Medicine, Aristotle University of Thessaloniki, 54636 Thessaloniki, Greece; st_roumeliotis@hotmail.com (S.R.); teleftheriadis@yahoo.com (T.E.)

**Keywords:** cosmic radiation, microgravity, kidney, oxidative stress, space

## Abstract

In space, the special conditions of hypogravity and exposure to cosmic radiation have substantial differences compared to terrestrial circumstances, and a multidimensional impact on the human body and human organ functions. Cosmic radiation provokes cellular and gene damage, and the generation of reactive oxygen species (ROS), leading to a dysregulation in the oxidants–antioxidants balance, and to the inflammatory response. Other practical factors contributing to these dysregulations in space environment include increased bone resorption, impaired anabolic response, and even difficulties in detecting oxidative stress in blood and urine samples. Enhanced oxidative stress affects mitochondrial and endothelial functions, contributes to reduced natriuresis and the development of hypertension, and may play an additive role in the formation of kidney stones. Finally, the composition of urine protein excretion is significantly altered, depicting possible tubular dysfunction.

## 1. Introduction

Space exploration draws a growing amount of attention not only for scientific purposes, but also for recreational travelling. This is mirrored in the efforts made to gain detailed knowledge on the special conditions that characterize space environment. In general, these conditions include weightlessness, exposure to cosmic radiation, hypogravity, and hyperoxia for astronauts [1]. The National Aeronautics and Space Administration (NASA) agency has introduced the Human System Risk Management as a means for a space travelling capability assessment for individuals who are interested in participating in space excursions [2].

Space radiation consists of galactic cosmic radiation (GCR) and solar cosmic radiation (SCR). The main components of GCR are baryons, protons, and alpha particles, and only less than 1% are heavy, high energy particles (HZE) like iron ions, which however mediate the biggest part of radiation received by cells even if they do not outweigh other GCR components. The main components of SCR are protons; SCR does not follow a constant pattern as is the case of GCR, resulting in fluctuating doses of radiation that are received by astronauts during extra-vehicular activities (EVAs) [3]. Immediately after take-off, spacecraft members have to face major accelerating forces and gravity change conditions that are simulated during training in parabolic flight conditions [4]. Nevertheless, those rapidly changing circumstances carry a significantly stressful burden for humans [5] which recently has been paralleled with the physiology of ageing [6]

### 1.1. The Induction of Oxidative Stress in Space: How The Story Starts

The radiation levels that humans are exposed at during spaceflights vary from 0.1–1 mGy/day, 100 times higher compared to Earth [7] (Table 1). The specifying of the biological impact of space radiation exposure on human organs remains vague [8] due to different tissue characteristics (location, volume) that influence absorption, as well as primary and secondary effects that are provoked by energy distribution [9]. Several other factors influence further radiation absorption, namely, spacecraft shielding, EVAs, human body orientation while in the spacecraft and more, complicating the evaluation of the cumulative radiation dose for astronauts during spaceflight [9]. Previously, in vitro experiments with epithelial cells have shown that HZE particles have the ability to cause oxidative stress in a dose-dependent manner, but at a lower magnitude compared to X-rays and γ-irradiation [10]. HZE particles are main space radiation constituent, and they have a critical significance in provoking the oxidative stress response, not only on the directly affected cells, but also to those in their vicinity. Reactive oxygen species (ROS) reactions in bystander cells were validated after the co-culture of irradiated cells with naive cells, resulting in the replication of equal effects such as oxidases and nitric oxide synthases activation, mitochondrial dysfunction, and genetic and epigenetic changes in both cell populations [11]. Microgravity is another oxidative stress promoter. Increased oxidative stress is particularly obvious after space flight, when damage products such as isoprostanes and 8-iso-deoxyguanosine are detected. A possible explanation for this phenomenon is the reduced metabolic rate during spaceflight that could be described as a “safe-mode” function, in order for the body to manage through low energy intake circumstances. This slowdown of metabolism, reducing even the production of pro-oxidant products, in combination with the loss of muscle and bone, form a negative energy balance. Muscle atrophy in space, as in other mechanical unloading circumstances, is caused by the activation of complex catabolic pathways, and it is further promoted in an environment of enhanced ROS production and altered calcium balance [12] Finally, the return to earth environment results in a restoration process that occurs through a burst of ROS production [13].

Oxidative stress in the environment of weightlessness and space irradiation has been shown in several tissue types like ocular tissue [14], neural stem cells [15], as well as brain cortex and hippocampus [16], skin [17], and intestine [18]. During the ESA-SPHINX (European Space Agency’s-SPaceflight of Huvec: an Integrated eXperiment) experiment, the induction of oxidative stress response was shown after studying the impact of space environment exposure on twelve cell-kits of human umbilical vein endothelial cells (HUVECS). Among the main findings of this experiment were the modification of the gene expression profile. In particular, there was a significant increase of *TXNIP* gene expression and its product, the inhibiting protein of the antioxidant thioredoxin [19]. Thioredoxin is a main regulator of redox status in endothelial cells [20]. The upregulation of the *TXNIP* gene has been correlated with enhanced inflammation, increased risk for coronary artery disease [21], and carcinogenesis [22]. Moreover, a negative impact on endothelial function during spaceflight was described regarding cytoskeletal integrity with alterations in integrins, actin-associated proteins, and adhesion molecules expression that further lead to impaired cell function. Additionally, OS expansion was shown through heat-shock protein downregulation, along with a change in cytokine profile, with increased levels of interleukin 1α and 1β. These alterations form the frame for endothelial dysfunction that could result in vascular ageing [19]. Similar degenerative processes have been described regarding microgravity and radiation effects on the cardiovascular system through NOX (NADPH oxidase) protein monomerization and the production of different types of ROS (especially superoxide anion) [23]. Therefore, according to available evidence, the significance of oxidative stress during spaceflight is multifactorial, results in severe endothelial damage, and has a multidimensional impact on space travelers with manifestations from several organs. Kidneys especially hold a primary role in space environment conditions, as they are both oxidative stress regulators and at the same time, highly vulnerable organs to oxidative damage (Table 2).

### 1.2. Fluid Balance in Space and Hypobaric Hypoxia

Body fluid regulation is remarkably changed in microgravity conditions, and it could be described as biphasic [24]. In an early in-flight acute phase, the reduced gravitational force leads to a shift of fluid into the thorax, and an increased central blood volume with the characteristic phenotype of “puffy face and birdy legs”. This phenomenon, according to the “Henry Gauer reflex theory” leads to the increased release of atrial natriuretic peptide (ANP) and consequently natriuresis, diuresis, and weight loss. [25]. However, the aforementioned theory was based on experimental models, and it was not confirmed during spaceflight. In parallel, central venous pressure is decreased, due to a reduced mediastinal pressure and to the increased thoracic cage compliance accompanying the diminished mechanical pressure on tissues and organs. Although a 10–17% reduction in plasma volume was documented, total body water and body weight did not seem to change [26]. The explanation could be based on the reduced transverse gravitational force that leads to a fluid redistribution from the intravascular space to the extravascular (intracellular and interstitial) [27,28]. At the same time, decreased renal sodium excretion has been observed, attributed mainly to an increase in sodium reabsorption due to the increased filtration fraction that accompanies the initial transient increase of GFR (glomerular filtration rate) [28,29]. Furthermore, due to space motion sickness and the enhanced pituitary release, ADH (anti-diuretic hormone) levels are initially very high [26].

In late in-flight chronic adaptation, as a new steady state is reached, ANP levels decrease, the sympathetic nervous system and the renin–angiotensin–aldosterone systems are activated, ADH remains elevated due to the decreased plasma volume, diuresis is reduced, and sodium is continuously retained, but it is redistributed in the extravascular compartment. The decoupling of renal sodium and water handling could be explained by persistent hypercalciuria due to the excessive bone mineral loss. Hypercalciuria, by promoting the proteolysis of the AQP-2 (aquaporin) water channel and stimulating CaSR (calcium sensing receptors) in the renal tubules, blunts the action of ADH in the collecting duct [30,31]. 

Hypobaric hypoxia is another component of the demanding space environment that the human body has to face, in which the oxygen concentration of the air is altered. Progress at multiple levels has been made regarding equipment (suits, vehicles, etc.), preparation, and recovery protocols, in order to eliminate decompression sickness [32,33]. In a hypoxic environment, the occurrence of reflex tachypnea, in order to increase alveolar oxygen, leads to hypocapnia and sustained respiratory alkalosis. The hypoxic diuretic response consists of the counterbalancing of renal excretion of excess bicarbonate, which is accompanied by increased natriuresis and water excretion. Stress hormones seem to participate in this response, suggesting the decreased sensitivity of the renal tubule to ADH, and decreased renal sympathetic nerve activity, further enhancing diuresis and sodium excretion. A new steady state seems to be established after 24 h, with bicarbonate excretion close to terrestrial environment levels [34]. However, the existence of a hypoxic diuretic response remains an issue of debate, while the majority of evidence comes from studies conducted in mountain climbers.

Hypobaric hypoxia and sodium retention have been linked with endothelial dysfunction and oxidative stress in terrestrial conditions. Oxygen deprivation leads to tissue hypoxia, and as an important promoter of oxidative stress, it consequently enhances endothelial dysfunction through impaired vasorelaxation and increased ROS production. Hypoxia is related to multiple organ damage, among which acute and chronic kidney injury through neurohormonal dysregulation and mitochondrial dysfunction apart from ROS increase [35]. On the other hand, sodium overload has been linked with impaired vasorelaxation and with endothelial dysfunction. Among possible proposed mechanisms are decreased nitric oxide release and increased superoxide anion production [36], even in the absence of elevated blood pressure [37]. These effects are triggered even shortly after sodium loading [38], a situation that can probably be paralleled with sodium handling in a space environment.

### 1.3. The Kidney, Mitochondria and Oxidative Stress

The induction of oxidative stress, first described in 1985 [39], equals the unsuccessful effort of a living organism to counterbalance a malignant stimulus that results in metabolic disturbances. Oxidative stress is characterized by the further dysregulation of the pro-oxidant and antioxidant equilibrium in favor of ROS production, such as superoxide anions (O_2_^−^), hydroxyl radicals (OH·) and hydrogen peroxide (H_2_O_2_) [40]. If this process is inadequately controlled by endogenous antioxidant systems, such as glutathione, coenzyme Q, uric acid, α-lipoic acid, melatonin, etc. [41], the progress of damage is inevitable, with ROS irrespectively targeting proteins, DNA, and lipids [40].

Oxygen is vital for aerobic organisms, and more than 90% is consumed in energy production (ATP) through oxidative phosphorylation in the electron transport chain (ETC), which is located on the apical side of the inner mitochondrial membrane. Part of the remaining oxygen (~4%) via ETC electron leakage, is reduced into O_2_^−^ and OH·, which, following their chemical trend, can detach hydrogen atoms from proteins, lipids, and enzymes in order to form water (H_2_O). This process leaves behind lipid, protein, and nucleic peroxides, which belong to the ROS family, adding on to the oxidative stress burden [40]. Kidneys receive about one-fourth of the total blood volume, maintaining an adequate corticomedullary oxygen concentration gradient in order to meet their needs and to fulfill their excretion, absorption, and neurohormonal functions. Moreover, kidneys can increase the blood supply under oxygen deprivation circumstances, implicating that they are remarkably rich in mitochondria [42]. The mitochondrion is a main source of oxidative stress generation, but it carries its own mechanisms to prevent damage: (1) endogenous antioxidants (glutathione, glutathione peroxidase); (2) the ability to open the mitochondrial permeability transition pore (MPTP) on demand, in order to speed up oxygen consumption and to restore electrochemical balance; and (3) mitophagy through the activation of PINK1/Parkin and the formation of autophagosomes that recycle useful compounds and isolate harmful compounds [43]. Mitochondrial pathology is related to a large spectrum of renal diseases, from glomerular (i.e., focal segmental glomerulosclerosis) and tubular diseases (mitochondrial tubulopathies, e.g., myoclonic epilepsy and ragged red muscle fibers (MERRF), Pearson’s, Kearns-Sayre, and Leigh syndromes) to structural, polycystic diseases [43]. When the initial stimuli that raise the oxidative stress response in the mitochondria fail to be controlled, the accelerating response leads to damage expansion, resulting in acute kidney injury. If malfunction is sustained, permanent damage varying from chronic kidney disease to end-stage kidney disease and even death is established [44].

### 1.4. Mitochondrial Changes after Radiation Exposure

After the application of space radiation, water, the most abundant cellular constituent, undergoes dissolution, leading to the formation of hydrated electron products (O_2_^−^, OH^−^ H_2_O_2_) resulting in an additional source of ROS production. This production is independent from the endogenous oxidative stress products. Moreover, secondary non-targeted alterations on gene multiplication, stability, expression, and rectifying processes take place, generating cell dysfunction and replication disturbances, neoplastic transformation, and even cell death [11]. It is noteworthy to comment that these secondary effects are of great significance, not only for their potential to cause cancer, but also for the effects on oxidative stress response induced by secondary space radiation (i.e., α particles, δ rays). Those bystander effects are important regarding the cellular damage and epigenetic alterations on neighboring cells that lead to an altered phenotype with multiple consequences [45]. 

The sequence of facts in an experimental model after the application of ionizing radiation on cells begins with a transient increase in ROS production lasting for a few minutes, followed by a decrease in the mitochondrial oxidation regulator, nicotinamide adenine dinoucleotide phosphate (NADPH) oxidase. After about 24 h, there is a peak of ROS levels and mitochondrial genome oxidation [46], showing that the damage process probably continues for days before the refuting process of ongoing OS damage and the antioxidant mechanisms’ counterbalancing actions are integrated. The final outcome could be either permanent impairment or repair. The HAIR experiment aimed for the quantification of mitochondrial (mt) to nuclear (n) DNA and RNA ratios, as well as the quantification of antioxidant levels before, during, and after a space mission. The authors found significant reductions in mtDNA and mtRNA during and after flight, and important diminutions in the mtRNA/nRNA ratios during-flight and in post-flight measurements. The mtRNA/mtDNA ratios remained relatively unchanged, with the exception of the post-flight samples. Moreover, authors investigated a number of antioxidants, and they found a significant reduction in the expression of all of the redox-related genes in post-flight samples. Only catalase levels did not decrease post-flight [47]. The observed decrease of mitochondrial genome expression is indicative of the damage that mitochondria undergo while in the oxidant environment of space, which may not ameliorate after spaceflight.

### 1.5. NADPH Oxidases, Endothelial Function, and Oxidative Stress in Space: What We Know about the Renal Response

Angiogenesis is fundamental for the survival of living organisms, in order to maintain sufficient blood, oxygen, and nutrient supplies to tissues, and to remove metabolic waste. Vascular formation is provoked by an inflammatory response [48], probably related to healing [49], or to tumorigenesis [31]. The correlation of angiogenesis with inflammation and their inseparable connection with the oxidative stress response, has been suggested and analyzed in several studies [50,51,52,53,54].

The main source of ROS production in the endothelium are NADPH oxidases (NOX), which seems to be their major function, with positive effects on cellular differentiation, cytoskeletal function, gene expression, and vascular tone regulation [55]. Low grade oxidative stress has beneficial impacts on the generation and maintenance of normal repair and growth pathways, and therefore antioxidant administration may inhibit not only the oxidation cascade, but also vascular renaissance [51]. ROS seems to upregulate angiogenesis, mainly through vascular endothelial growth factor (VEGF) pathways [38], but also via lipid-damage products, such as the carboxylalkyl pyrrole (CAP) proteins that share similar angiogenetic effects with VEGF in in vitro experiments [56]. NADPH oxidases hold a critical role on the pathophysiology and progression of atherosclerosis and cardiovascular disease [57,58]. 

The mechanisms behind endothelial damage after ionizing radiation exposure have not been fully elucidated yet, with the majority of evidence coming from experimental models in oncology and radiotherapy. DNA impairment after radiation exposure leads to the upregulation of transduction cascades such as the *p53*/*p21* pathway, which through the increase of *p16*, results in cell cycle arrest [59]. This protective response for the elimination of abnormal cells is broadly called senescence. Insufficient DNA repair after radiation damage, along with the minimization of vascular regeneration, have been shown [60]. In the same study, the acquisition of the senescence-associated secretory phenotype (SASP) was observed [60], which is indicative of the subsequent generated pro-inflammatory process potentially leading to tumorigenesis [61]. Another in vitro study showed secondarily induced DNA damage from ROS production, and not as a direct impact of radiation [62], adding to the complexity of mechanisms underlying the general impact of radiation on cellular integrity. 

In an experimental model with ionizing irradiation on cardiac microvascular endothelial cells, apart from the dysregulation of cytoskeletal protein levels (i.e., vimentin, tubulin, actin, intracellular adhesion molecules (ICAMs)), impaired vasodilation was also observed, due to the decrease of nitric oxide [NO] [63]. This is in agreement with increased NO degradation from NOX-derived ROS, through the eNOS uncoupling phenomenon [55,64]. This phenomenon leads to prolonged vasoconstriction, attenuation of oxygen delivery, and consequent tissue hypoxia, which may result in acute or chronic organ damage. Results from the same study showed the increase of a number of oxidative stress products, such as oxidized low-density lipoproteins and heat shock proteins, and the increase of inflammatory cytokines such as TNF-a, IL-1, and IL-6. Additionally, the study showed the distortion of the insulin/IGF-PI3K-Akt cascade that normally contributes to vasorelaxation through the production of endothelial NO [63]. The meeting of the obvious need for more well-designed studies focusing on acute organ damage from cosmic radiation will add on to the comprehension of space environment impacts on humans.

Proximal tubule Na^+^/K^+^-ATPase seems to be upregulated by superoxide, enhancing sodium absorption, while an increase of filtration fluid volume in the thick ascending limb of the Henle loop provokes ROS generation. This further stimulates Na^+^/K^+^/2Cl^−^ cotransporters, increasing the total sodium load [65]. While in the terrestrial environment, the sodium balance is maintained by the “safety valves” of the tubules, i.e., tubuloglomerular feedback and glomerotubular balance [66], circumstances may differ under the impact of microgravity. The altered fluid distribution when weightlessness is present leads to complex hemodynamic dysregulations that affect total body water and sodium. A single astronaut observation during space flight revealed a constant reduction in natriuresis and diuresis rate that refuted the earlier concept of fluid loss as a contributor to the observed weight loss post-flight [67]. In a study of four astronauts, data from flight were compared to ground evidence, and there was an increase of urodilatin, the main renal natriuretic peptide [68], after an initially observed decrease during the first 24 h [69]. After a saline infusion challenge during the fifth day, when a new balance in microgravity was well established, the renal response regarding natriuresis and urodilatin levels in the urine was attenuated compared to earth data, though the reaction lasted for a longer time period in space [69]. This result probably indicates that the integrity of the regulatory mechanisms is maintained in microgravity, and other contributing factors could affect the final outcome. In another study, authors investigated the kinetics of body mass changes during two space flights (EuroMIR94, 30 days and MIR97, 19 days). In particular, three astronauts were monitored with regard to their diet in the first mission, while in the second mission, astronauts were under a predefined constant energy and sodium intake. Evidence from the MIR97 missions showed that a significant reduction in natriuresis under the predefined sodium intake resulted in the accumulation of 17g of sodium after two weeks in flight, with no simultaneous change in urine osmolality [70].

### 1.6. NADPH Oxidases in the Pathophysiology of Hypertension: How Does the Story Go in Space?

From the seven members of the NOX family, the main representative in the kidney is NOX4, followed by NOX2; both are found in several segments throughout the renal tubule, vascular stroma and parenchyma [71]. Superoxide anions produced by NOX enhance the induction of tubular cell gluconeogenesis, as well as the transport of glucose through sodium/glucose cotransporters (SGLT2) [65], and the role of NOX in the pathophysiology of diabetes is under research [72]. 

NOX4 enhances vasorelaxation by increasing NO release via hydrogen hyperoxide production, which through the oxidation of G1a kinase, promotes vascular dilatation. On the other hand, the increase in superoxide produced by NOX1 and NOX2 leads to deficient levels of NO, and endothelial dysfunction. NOX1 and NOX2, together with their subunits, p47phox and p22phox, seem to be involved in the pathophysiology of hypertension [73]. The participation of NOX1 and NOX2 in hypertension is supported by experimental models that show a significant increase of NOX1 expression in vascular smooth muscle cells (VSMC) in hypertensive models [74]. On the other hand, the elimination of NOX2 from the vasculature rendered subjects relatively unaffected by angiotensin II [75]. Angiotensin exerts its action through the activation of the AT1-receptor in VSMC, leading to NOX1 increase. This increase translates into superoxide upregulation, phosphorylation of serine/threonine kinases (p38MAPK, Akt, ERK1/2), vascular senescence, and mitochondrial dysfunction, through the acetylation of the PGC-1a transcription factor by angiotensin II [76]. NADPH oxidases and their subunits have been broadly studied through several model designs (separately and in combination), indicating that they are not only related to the development of hypertension, but also with vascular remodeling and the progression to vascular stiffness through a net of mechanisms where ROS and oxidation process hold the central role [73,77]. 

Experimental models show increased NOX activity on renal cortex after the exogenous administration of angiotensin II [75,78], which is diminished after treatment with antioxidants such as superoxide dismutase (SOD) [79]. The elimination of NOX2 has been shown to attenuate the renal vasoconstriction response to angiotensin II [80], supporting the theory that angiotensin II action on renal vasculature is mediated by superoxide anion. A diet rich in sodium chloride leads to an increased activation of NOX on the renal cortex, and to a decrease of SOD [81]. Subsequent blood pressure dysregulation occurs in the setting of increased oxidative stress, irrespective of the reduction of renin and angiotensin levels [71]. Kidneys are characterized by a rich vascular net, and they are vulnerable to acute and chronic damage in the presence of endothelial dysfunction and oxidative stress. As discussed earlier, during spaceflight and EVAs, the human body is exposed to a hostile environment with radiation and microgravity-induced oxidative stress, electrolyte and fluid dysregulation, skeletal and total body mass loss, and endothelial dysfunction. According to theory, all these parameters in combination with the low ANP levels, possibly further abrogated by the gradual magnesium deficiency, and with an enhanced catecholamine profile, lead to sustained renal artery vasoconstriction. This predisposes the individual to renovascular hypertension, as well as permanent glomerular damage [82]. 

A small number of experimental and human studies however, show results that contradict the former theory. A rat model investigated the effect of chronic intermittent hypobaric hypoxia (CIHH) on arterial blood pressure (ABP) and baroreflex function in renal vascular hypertension (RVH). According to the model, CIHH resulted in the recession of renovascular hypertension through the facilitation of a baroreflex control [83]. Moreover, the beneficial impact of radiation was shown in nephrectomized rats that achieved better blood pressure control and proteinuria levels, compared to their controls [84]. Earlier observations suggest a reduction in both diastolic blood pressure and heart rate in a microgravity environment [85], while the initial increase in systolic blood pressure observed during a space stay was downregulated after a couple of weeks to preflight levels, and it was accompanied by a new motive in blood pressure control during space travel [86]. 

### 1.7. Space and Other Kidney-Related Manifestations

Nephrolithiasis is a recognized problem for astronauts, and as shown from previous studies, the probability of stone formation in a microgravity environment is higher than on Earth, including even post-flight [87] (Table 3). A prediction model has been introduced that estimates the risk for kidney stone formation in space travelers with and without prior nephrolithiasis, using biochemical evidence from 24 h urine collections [88]. This study group has evaluated their own model in a small sample, and has confirmed the already-observed depletion of calcium and oxalate ions from urine after stone formation, which are attributed to the enlargement of a newly formed stone [88]. Calcium-sensing receptors (CasR) exist in several segments throughout the renal tubule, and they play an important role in calcium level regulation. Mutations of CasR are related to electrolyte and water management dysregulations [89]. Aquaporins are known to participate in water balance as renal water excretion channels. Their possible role in further attenuating the reduction of urine output in the hypercalcemic astronaut has been suggested, along with the possible different effect that CasRs might pose on aquaporin 2 expression during spaceflight [31]. This effect may not result in the expected downregulation of aquaporin 2 by CasRs activation, because of the additional impact of increased ADH in a microgravity environment that further leads to water reabsorption through aquaporin 2 upregulation [90].

The most common composition of kidney stones is that of calcium oxalate, with excess calcium resulting from bone mobilization in space, and in the concomitant presence of urine hypersaturation. Contributing factors to the formation and further growth of renal calculi are hypocitruria, hyperoxaluria, pH reduction, and dietary parameters such as increased protein consumption, decreased magnesium intake, and mainly reduced hydration [91], which leads to a concentrated urine output. The analysis of 23 crewmembers’ data showed that apart from bone resorption, an increased risk for renal stone formation was present, irrespective of the exercise undertaken. The main contributor to nephrolithiasis was decreased urine volume with a high concentration of oxalate, calcium, and brushite [92]. In a study of 42 astronauts (33 men and nine women) no sex difference was proven regarding bone loss, urine hypersaturation, and risk of renal stone formation [93]. The colonization of the human body with nanobacteria has been suggested to be a predisposing factor for renal calculi formation in the microgravity environment, through their ability to induce calcium phosphate precipitation [94]. It has been shown that in simulated microgravity conditions the nanobacteria multiplication rate is increased by up to 4.6 times compared to the terrestrial environment, but the possible significance of pre-flight identification of nanobacteria flora remains to be defined [95]. Recent data from a study of 30 patients with nephrolithiasis showed that nanobacteria were present in all 27 patients with apatite kidney stones, supporting their correlation with renal stone formation [96]. Preventive measures of stone formation besides hydration, include alkali (i.e., citrate) and pyrophosphate supplementation [97,98]. The presence of renal stones provokes a significant OS response in renal cells through the increase of superoxide anion [99], and increased urine DNA damage biomarkers, i.e., 8-hydroxydeoxyguanosine [100]. However, more data are needed in order to better clarify the underlining mechanisms.

Urine albumin excretion in normal subjects is low (<30 mg/day), due to albumin’s charge and size and its tubular reabsorption. With any dysregulations to glomerular filtration and to the tubular barrier, which vary from transient stressor effects to established effects, systemic or local vascular damage may result in consistent microalbuminuria [101]. Microalbuminuria has been recognized as a major risk factor for cardiovascular disease [102], along with the therapeutic importance of the reduction of urine protein excretion through renin–angiotensin axis inhibition [85]. According to the available data, sustained oxidative stress correlates with albuminuria through glomerular, tubular, and vascular damage caused by ROS [103,104,105,106]. Observational data in astronauts shows a reduction of urine albumin excretion during space missions, compared to pre- and post-flight, which remains only partly explained [107]. Hemodynamic changes and body fluids re-distribution in microgravity conditions seem to participate in this phenomenon [31]. Efforts have been made to analyze the proteomic content of urine during spaceflight, in order to study the impact of microgravity in its composition. Gonzales et al., compared urine protein composition before and after space flight in 10 cosmonauts, focusing on tissue origin, and found 238 proteins, among which there were proteins that were regularly found in urine, such as cubulin, megalin and uromodulin [108]. Three other proteins were detected only post-flight, afamin, aminopeptidase A, and aquaporin 2. Afamin has an antioxidant function, is upregulated by hypercalcemia, but may also come from the lower urinary tract. Aminopeptidase A is a marker of renal tissue hypoxia, and it is upregulated in tubular dysfunction. Aquaporin 2, with its well-known role in renal water excretion, is located on the apical side throughout the tubule. Higher levels of afamin and aquaporin 2 were detected after flight, a finding that further enhances ideas on their participation in electrolyte balance, especially calcium and water balance restoration [108]. Furthermore, all three proteins are indicative of a stressful response. Larina et al., used a simulated space environment to study the urine proteomics of the six participants during a 520-day follow-up, with seven measurements during that period and a single post-flight measurement, from which seven proteins out of the 256 identified in total remained constant. After further analysis according to tissue origin, gene encoding, and action, no correlations were found for these proteins. Nevertheless, a biomarker of tubular dysfunction, the precursor of a1-micoglobulin, as well as a plasma serine protease inhibitor that is also found in renal tubular cells, were identified [109]. One of the limitations of the published study is that the levels of the identified proteins are not provided and no discussion is made. No studies exist regarding the progress of these findings in the long run, and their impact on the manifestation of clinically significant renal disease.

## 2. Conclusions

Space medicine is an expanding field with remarkably interesting findings on human function alterations during exposure to microgravity and cosmic radiation. Accumulating knowledge has already begun to be analyzed, in order to improve our comprehension on pathogenetic mechanisms of several degenerative diseases and tumorigenesis. However, limitations still exist regarding the study of organ exposure to radiation, as the generation of an accurate prediction model is affected by multiple parameters.

Knowledge of the renal response during space missions remains limited. The impact on kidney function is multifactorial, and besides, microgravity and radiation exposure includes altered caloric and hydration intake compared to terrestrial circumstances, as well as multiple neurohormonal changes. The established enhanced oxidative stress environment during and after space flights and the subsequent mitochondrial dysfunction caused by radiation exposure leads to kidney damage at multiple levels, affecting both tubular and glomerular integrity, and micro- and macrovascular function. Indications from the available data suggest that the kidney undergoes a severe oxidative stress attack during spaceflight, as mirrored by studies that detect oxidation products in urine. However, the actual long-term impact on kidney function post-spaceflight remains undetermined. Further investigation is needed regarding the impact of space flight on renal survival and possible glomerular dysfunction.

## Figures and Tables

**Table 1:** Studies investigating the possible link between space environment conditions with functional and regulatory organ and tissue mechanisms.

	Year	Type of Study	Study Facilities/Environment	Duration	Subject Characteristics	Methods	Main Findings
^10^Wan, S.; et al.	2005	In Vitro	Laboratory facility		Human breast cells	Radiation (γ-, X-rays, protons, HZE particles, ^56^Fe ions)	OS correlated with radiation in a dose-depended pattern. HZE particle radiation was less effective in producing OS compared to γ- and Χ-rays
^19^Versari, et al.	2013	In Vitro	Spaceflight	10 days	Human umbilical vein endothelial cells (HUVECs)	Post-flight microarray gene analysis	1023 significantly modulated genes. Thioredoxin was the most highly upregulated. Heat-shock protein 90 and 70 were the most downregulated. Significant increase of IL-1a and IL-1β.
^47^Indo, et al.	2016	Cohort study	Spaceflight, post-flight	6 months	Hair samples by astronauts	Quantitative PCR for mtDNA/nDNA and mtRNA/nRNA. Antioxidant related gene expression (MnSOD, CuZnSOD, Nrf2, Keap1, GPx4 and Catalase)	Significant reduction of mtDNA/nDNA ratio during flight. Significant reductions in the mtRNA/nRNA ratios in inflight and post-flight samples. All antioxidant related genes’ expression was significantly decreased. Catalase gene expression remained unchanged.
^69^Drummer C, et al.	1997	Observational study	Space mission	1 week	Male astronaut	Saliva and urine sampling on prespecified time points.	Reduction of natriuresis and diuresis throughout the flight. Urodilatin excretion correlated with sodium excretion.
^71^Drummer C, et al.	1997	Observational study	Space mission	5 days	4 male astronauts	Urine and blood samples on prespecified time points (plus before 1.6L isotonic saline infusion).	ANP and cGMP levels did not increase after the infusion of saline (simulated plasma expansion). Renal excretion of urodilatin increased for several hours post saline infusion.
^72^Drummer, et al.	2000	Observational study	Space missions (EuroMIR94 and MIR97)	30 and 17 days	3 astronauts	Diet monitoring. Water and sodium balance. Body mass measurement. Blood samples.	Body weight loss is attributed to reduced caloric intake during spaceflight. Microgravity provokes sodium retention through hormonal dysregulations.
^87^Fritsch-Yelle, J.; et al.	1996	Observational study	Space missions	5 to 10 days	12 male astronauts	Blood pressure and heart rate monitoring during prespecified intervals.	Significant reduction of heart rate, diastolic blood pressure and premature ventricular contractions during spaceflight.
^88^Shiraishi, et al.	2004	Observational study	Space mission (MIR)	6 months	4 male astronauts	Blood pressure and heart rate measurement.	Systolic blood pressure during sleep on spaceflight were increased compared to earth. The initially prolonged periodicity of blood pressure and heart rate was shortened to pre-flight values after a few months on space mission.

OS: oxidative stress, ROS: reactive oxygen species, IL: interleukin, mt: mitochondrial, n: nuclear, ANP: atrial natriuretic peptide, cGMP: cyclic guanosine monophosphate, mnSOD: manganese superoxide dismutase.

**Table 2:** Main aspects of space impact on renal function.

ROS generation ▪ HZE particles exposure ▪ Microgravity
Mitochondrial dysfunction
Endothelial dysfunction
Vascular senescence
Non-targeted effects (on genes’ stability, expression, replication)
Hemodynamic changes ▪ Fluid distribution ▪ Neurohormonal balance
Nephrolithiasis
ADH levels alteration
Hypercalciuria/hypocitraturia
Sodium handling

HZE: high energy, ADH: antidiuretic hormone, ROS: reactive oxygen species.

**Table 3:** Studies investigating renal stone formation in the space environment.

Authors	Year	Type of Study	Study Facilities/Environment	Duration	Subject Characteristics	Methods	Main Findings
^95^Smith, et al.	2015	Retrospective analysis	Space missions	Variable (>100 days)	23 astronauts (four women) Seven astronauts received aledronate (six men, one woman)	Blood and urine samples (pre and during spaceflight). Resistive exercise equipment.	Risk of nephrolithiasis increased during spaceflight (irrespectively to exercise intensity) in all groups. Significant increase of sclerostin during spaceflight in all groups. Urine supersaturation risk was higher compared to the general population.
^96^Smith, et al.	2014	Retrospec tive analysis	Space missions	Variable (49–215 days)	42 astronauts (33 men and nine women)	Blood and urine samples pre and post flight. Bone densitometry. Resistive exercise equipment.	No sex difference in the response of bone mineral density. Equal risk for urine supersaturation between sexes. Equal risk for stone formation between sexes.
^98^Ciftcioglu, et al.	2005	In vitro study	High aspect rotation vessels (HARV)—microgravity simulation. Stationary and shaker cultures.	Non applicable	Nanobacteria cultures	Spectrophotometer analysis, SEM, TEM, EDX.	4.6 times faster multiplication of nanobacteria in HARVs. Existence of apatite crystals on all nanobacteria cultures. Thinner layer of apatite on HARV cultures.

SEM: scanning electron microscopy, TEM: transmission electron microscopic analysis, EDX: energy-dispersive X-ray analysis.
